# Brain Volume in Fetal Alcohol Spectrum Disorders Over a 20-Year Span

**DOI:** 10.1001/jamanetworkopen.2023.43618

**Published:** 2023-11-17

**Authors:** Adolf Pfefferbaum, Edith V. Sullivan, Kilian M. Pohl, Amanda Bischoff-Grethe, Susan A. Stoner, Eileen M. Moore, Edward P. Riley

**Affiliations:** 1 Center for Health Sciences, SRI International, Menlo Park, California; 2 Department of Psychiatry and Behavioral Sciences, Stanford University School of Medicine, Stanford, California; 3 Department of Psychiatry, University of California, San Diego, La Jolla; 4 Department of Psychiatry and Behavioral Sciences, University of Washington, Seattle; 5 Department of Psychology, San Diego State University, San Diego, California

## Abstract

**Importance:**

Anomalous brain development and mental health problems are prevalent in fetal alcohol spectrum disorders (FASD), but there is a paucity of longitudinal brain imaging research into adulthood. This study presents long-term follow-up of brain volumetrics in a cohort of participants with FASD.

**Objective:**

To test whether brain tissue declines faster with aging in individuals with FASD compared with control participants.

**Design, Setting, and Participants:**

This cohort study used magnetic resonance imaging (MRI) data collected from individuals with FASD and control individuals (age 13-37 years at first magnetic resonance imaging [MRI1] acquired 1997-2000) compared with data collected 20 years later (MRI2; 2018-2021). Participants were recruited for MRI1 through the University of Washington Fetal Alcohol Syndrome (FAS) Follow-Up Study. For MRI2, former participants were recruited by the University of Washington Fetal Alcohol and Drug Unit. Data were analyzed from October 2022 to August 2023.

**Main Outcomes and Measures:**

Intracranial volume (ICV) and regional cortical and cerebellar gray matter, white matter, and cerebrospinal fluid volumes were quantified automatically and analyzed, with group and sex as between-participant factors and age as a within-participant variable.

**Results:**

Of 174 individuals with MRI1 data, 48 refused participation, 36 were unavailable, and 24 could not be located. The remaining 66 individuals (37.9%) were rescanned for MRI2, including 26 controls, 18 individuals with nondysmorphic heavily exposed fetal alcohol effects (FAE; diagnosed prior to MRI1), and 22 individuals with FAS. Mean (SD) age was 22.9 (5.6) years at MRI1 and 44.7 (6.5) years at MRI2, and 35 participants (53%) were male. The FAE and FAS groups exhibited enduring stepped volume deficits at MRI1 and MRI2; volumes among control participants were greater than among participants with FAE, which were greater than volumes among participants with FAS (eg, mean [SD] ICV: control, 1462.3 [119.3] cc at MRI1 and 1465.4 [129.4] cc at MRI2; FAE, 1375.6 [134.1] cc at MRI1 and 1371.7 [120.3] cc at MRI2; FAS, 1297.3 [163.0] cc at MRI1 and 1292.7 [172.1] cc at MRI2), without diagnosis-by-age interactions. Despite these persistent volume deficits, the FAE participants and FAS participants showed patterns of neurodevelopment within reference ranges: increase in white matter and decrease in gray matter of the cortex and decrease in white matter and increase in gray matter of the cerebellum.

**Conclusions and Relevance:**

The findings of this cohort study support a nonaccelerating enduring, brain structural dysmorphic spectrum following prenatal alcohol exposure and a diagnostic distinction based on the degree of dysmorphia. FASD was not a progressive brain structural disorder by middle age, but whether accelerated decline occurs in later years remains to be determined.

## Key Points

**Question:**

Do cortical volumes decline faster in individuals with a fetal alcohol spectrum disorders (FASD) than unaffected controls over 20 years from adolescence to middle age?

**Findings:**

This cohort study among 66 individuals, including 26 healthy controls, 18 individuals with fetal alcohol effects, and 22 individuals with fetal alcohol syndrome found that cortical volumes of the FASD groups declined at the same rate as observed in controls despite persistent volume deficits graded by diagnostic severity.

**Meaning:**

These findings suggest that a sustained spectrum of brain volume deficits endured from adolescence into early middle age based on the original severity of dysmorphia and a diagnostic distinction between fetal alcohol syndrome and alcohol-related neurodevelopment disorder.

## Teaser

This cohort studies uses magnetic resonance images taken 20 years apart to assess whether brain tissue declines faster with aging in individuals with fetal alcohol spectrum disorders compared with unexposed controls.

## Introduction

Fetal alcohol spectrum disorders (FASD) are a collection of physical, neural, cognitive, and behavioral anomalies that can result from prenatal alcohol exposure (PAE).^1,2^ Seminal magnetic resonance imaging (MRI) studies of individuals with FASD have found intracranial volume (ICV) deficits compared with unexposed controls and regional deficits in the size of the basal ganglia, thalamus, hippocampus, corpus callosum, and cerebellum,^1^ with the latter 2 regions especially sensitive to PAE.^3,4,5,6^ Functional ramifications of regional neurodysmorphia in FASD^7,8^ span sensory,^9,10,11,12^ motor,^13,14^ and cognitive^15,16,17^ skills. Recent automated volumetry of legacy MRI data on FASD and control groups of individuals aged 13 to 30 years identified volume deficits in the corpus callosum, caudate, putamen, pallidum, and cerebellum in the FASD group compared with controls, even after controlling for ICV differences.^18^ Measurement of anatomically distinct lobules of the cerebellum revealed deficits in several lobules with the degree of deficit being related to the degree of dysmorphia.^19^

Although FASD is considered a lifelong disorder, little is known about this condition in adults^20^ because most FASD studies report solely on children and adolescents. The few MRI investigations in young adults are cross-sectional and note smaller volumes in brain areas consistent with those reported in youth.^21,22,23^ Whether the structural anomalies detected in FASD are progressive with aging has received little attention,^18^ with most research on FASD ending by age 30 years.^20,24^ The few extant longitudinal imaging studies have spanned only 2 to 5 years and focused on youth. For example, a study of individuals with FASD and controls aged 5.7 to 15.9 years at initial MRI found that white matter volumes increased linearly over 1.8 years in both groups, with no evidence for differences in growth trajectories of total brain volume or gray and white matter.^25^ A later comparison of individuals with FASD and controls followed up for 2 to 4 years found the FASD group had less cortical thinning than controls, suggesting delayed development.^26^ A 2-year follow-up study of youth with PAE and controls aged 6 to 17 years at the first MRI found a change in cortical thickness but not cortical volume in the PAE group.^27^

Given the paucity of longitudinal studies of FASD beyond emerging adulthood and the possibility of accelerated brain aging, as seen in other developmental disabilities,^28,29^ we conducted a quantitative analysis of MRI data from participants with FASD and unexposed control participants in the original University of Washington cohort who were rescanned a mean of 20 years after their initial MRI. Accordingly, the current study tested the following 3 hypotheses: (1) follow-up MRI data would evidence stepped deficits in cerebral and cerebellar volumes as observed with the initial MRI, where regional volumes by group would be largest in the control group, smaller in the fetal alcohol effects (FAE) group, and smallest in the fetal alcohol syndrome (FAS) group; (2) all groups would show the patterns of age-related brain volume changes within reference ranges, where gray matter shrinks while white matter expands over the 20-year interval spanning years of active neurodevelopment^30,31,32^; and (3) volume deficits of the FAE and FAS groups would show greater age-related declines relative to the control group regardless of sex.

## Methods

This study was approved by the institutional review board of the University of Washington. Informed consent was obtained according to the Declaration of Helsinki from adult participants or their legal guardians, in which case assent was obtained from the participant. This study is reported following the Strengthening the Reporting of Observational Studies in Epidemiology (STROBE) reporting guideline for cohort studies. An abstract for this study has been published elsewhere.^33^

### Participants

Of 59 control participants, 58 participants with FAE, and 57 participants with FAS in our initial report,^19^ 66 (37.9%) had useable follow-up MRIs: 26 control participants, 18 participants with FAE, and 22 participants with FAS. MRIs were acquired a mean (SD) of 21.6 (1.8) years after the first scan for the control group, 21.7 (1.8) years for the FAE group, and 20.6 (2.2) years for the FAS group. Patient ascertainment was from the Seattle FAS Follow-up Database, accrued over nearly 3 decades from referrals from or to dysmorphologists. Race was determined by self-identification or by identification by the physician as African American, American Indian or Alaskan Native, or White. Race was included only as a descriptor of the participants.

As described previously,^19^ participants were identified, recruited, clinically and neuropsychologically examined, and diagnosed for their first MRI (MRI1) through the FAS Follow-Up Study at the University of Washington.^3,34,35,36^ Of participants with FASD, systematic diagnosis used 1994 criteria to determine FAS and FAE. FAS required evidence of central nervous system dysfunction, growth deficit, and the presence of sentinel facial features, whereas FAE was classified when the full set of physical characteristics was not present in an individual with heavy PAE.^3^ Two sets of MRI data are reported in this study: the initial data set (MRI1), which was collected between 1997 and 2000, and the second data set (MRI2), which was collected between 2018 and 2021.

Former study participants were located using provided contact details and online resources to obtain current information and assess willingness to participate. Potential participants were contacted to explain the study and assess willingness to participate. Numerous other reports based on the full or partial cohort have been published.^3,18^

Exclusion criteria at the initial MRI were HIV infection, neurotoxic medications, legal blindness, dental braces, psychological testing within the past year, or not having English as their primary language. Control participants were excluded for alcohol or drug problems, neurological problems, birth defects involving the brain, self-reported auditory or visual hallucinations, having a bachelor’s degree or higher education, or having a biological mother with a reported history of substance abuse problems, particularly binge drinking around the time of their pregnancy with the individual.^3^

### MRI Acquisition and Analysis

MRI analysis was performed by computer, without supervision, and blind to diagnosis. After processing, images were reviewed blind for quantification quality. Native T1-weighted MRI legacy data at the first scan were acquired on a 1.5T Signa system (GE). Follow-up MRIs were acquired on a Ingenia Medical System 3T scanner (Philips).

**Figure 1.**
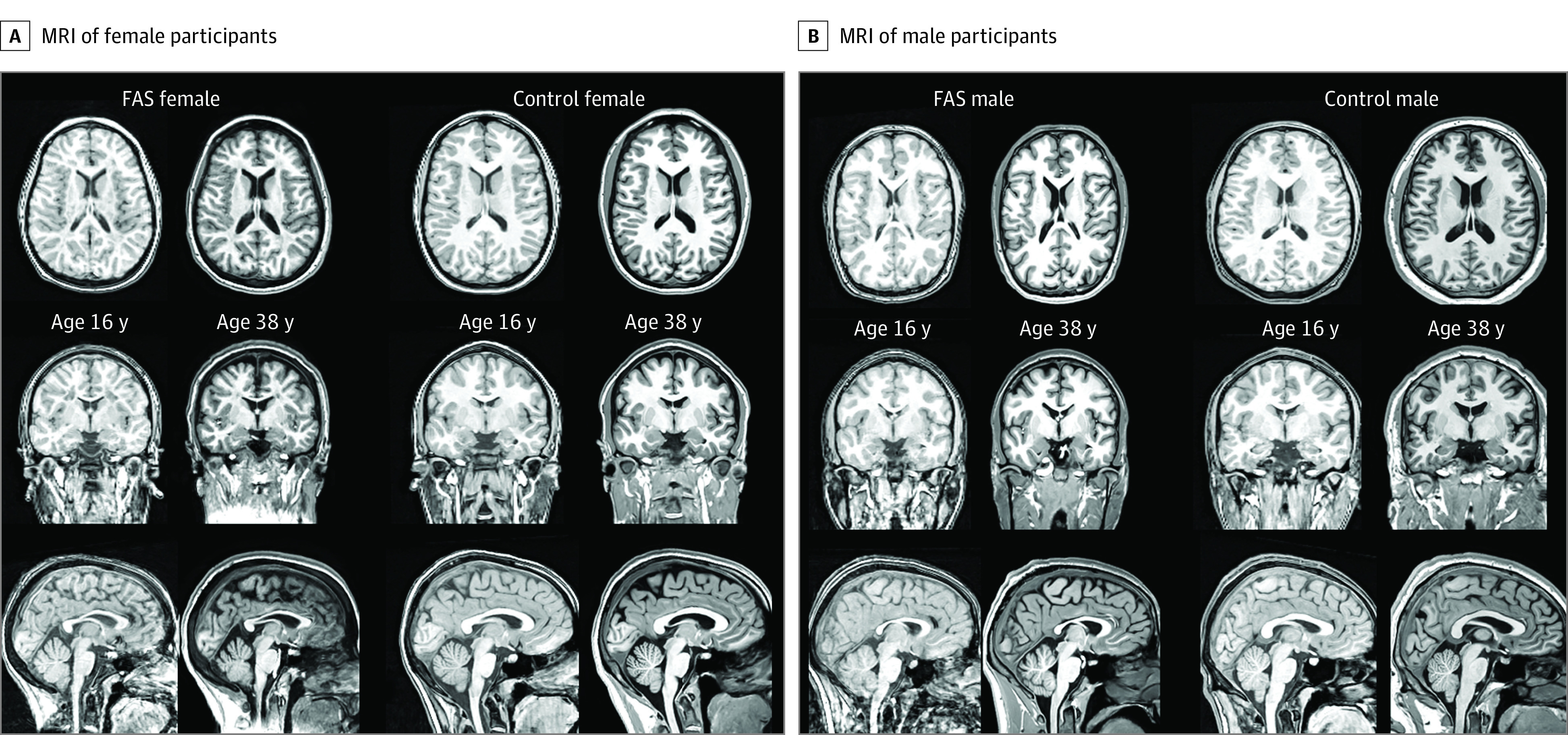
Examples of Magnetic Resonance Images (MRI) 1 and 2 in Participants With Fetal Alcohol Syndrome (FAS) and Healthy Controls From top to bottom, axial, coronal, and sagittal views of exemplary MRIs of participants at their initial and follow-up sessions approximately 20 years later. Note the enlarging of the lateral ventricles in both participants with FAS.

Because the original and follow-up scans differed in resolution and tissue conspicuity, the scans of a participant were analyzed together via Scalable Informatics for Biomedical Imaging Studies (SIBIS),^31^ a pipeline for the longitudinally consistent processing of brain MRIs (pairs of longitudinal scans shown in Figure 1A and B). For each participant, SIBIS first inhomogeneity corrected^32^ the higher-resolution follow-up scans and afterward produced a brain mask. The pipeline nonrigidly aligned the SRI24 atlas^37^ to the skull-stripped scan via Advanced Normalization Tools^38^ and used the aligned atlas to guide the tissue segmentation via Atropos^39^ and the parcellation of the cerebellum via a spatially unbiased infratentorial template.^40^ Next, the baseline scan was image-inhomogeneity corrected and rigidly registered to the follow-up scan. The resulting transformation was used to align the brain mask of the follow-up scan and the SRI24 atlas to the baseline. The pipeline repeated tissue and cerebellum segmentation. For baseline and follow-up, the pipeline determined the ICV and gray and white matter cortical and cerebellar regional volumes.^19^

### Statistical Analysis

Statistics were performed using R statistical software version 3.5.1 (R Project for Statistical Computing) on bilateral volumes, which were the sums of left and right regional volumes. The primary statistic for comparisons between returners and nonreturners was a general linear model (*lm*). The primary statistic for longitudinal analysis was a linear mixed effects model (*lmer*), which separately estimated the volume of each region as a function of diagnostic group (control, FAE, FAS) + ICV + sex + age. The mean value of the controls was added to the individual’s residual for plotting on a meaningful scale. Interactions were examined with 2-way (group-by-sex and group-by-age) and 3-way (group-by-sex-by-age) interactions. Interactions by age tested potential group differences in rates of change from the original to the follow-up scan. The model outputs produced *t* and *P* significance values for each diagnostic group compared with the controls. To compare FAE vs FAS groups, the model was rerun with FAS as the index level. Familywise Bonferroni corrections were applied to adjust *P* values to account for multiple comparisons. *P* values were 2-sided, and statistical significance was set at *P* = .05. Data were analyzed from October 2022 to August 2023.

## Results

Of 174 individuals with MRI1 data, 48 refused participation, 36 were unavailable, and 24 could not be located. The remaining 66 individuals (37.9%) were rescanned for MRI2, including 26 controls, 18 individuals with nondysmorphic heavily exposed fetal alcohol effects (diagnosed before MRI1), and 22 individuals with FAS. Mean (SD) age was 22.9 (5.6) years at MRI1 and 44.7 (6.5) years at MRI2, and 35 participants (53%) were male (Table 1).

**Table 1.** Participant Demographic Characteristic for the 3 Groups

Characteristic	Mean (SD)			Simple estimates by lm^a^				3-Group comparison	
	Control (n = 26)	FAE (n = 18)	FAS (n = 22)	Control vs FAE		Control vs FAS		χ^2^	*P* value
				*t*	*P* value	*t *	*P* value		
Age at MRI1, y									
Male	21.3 (6.8)	22.8 (6.2)	21.3 (6.8)	0.63	.53	−0.05	.96	NA	NA
Female	19.1 (5.6)	19.4 (5.8)	19.1 (5.6)						
Total	20.2 (6.1)	21.3 (6.1)	20.1 (4.7)						
Age at MRI2, y									
Male	43.4 (8.0)	45.4 (6.8)	41.2 (6.3)	0.58	.57	−0.49	.63	NA	NA
Female	40.4 (6.2)	40.1 (5.8)	40.7 (5.3)						
Total	41.9 (7.2)	43.1 (6.7)	41.0 (5.8)						
Race, No. (%)									
African American	1 (4)	1 (6)	2 (9)	NA	NA	NA	NA	1.714	.79
American Indian or Alaskan Native	4 (1)	5 (28)	4 (18)	NA	NA	NA	NA		
White	21 (81)	12 (67)	16 (73)	NA	NA	NA	NA		
Handedness, No.									
Right	25	15	14^b^	NA	NA	NA	NA	7.186	.03^a^
Left	1	3	7						
BMI at MRI 2	27.4 (5.2)	31.2 (7.7)^b^	27.3 (6.8)	1.92	.06	−0.06	.95	NA	NA
Education at MRI1, y	12.0 (2.7)	11.1 (1.9)	10.3 (2.2)	−1.26	.21	−2.43	.02	NA	NA
Wechsler Adult Intelligence Scale at MRI1									
Verbal IQ prorated	112.0 (10.6)	89.1 (11.5)	82.2 (11.4)^b^	−6.73	<.001	−9.13	<.001	NA	NA
Performance IQ	113.1 (14.5)	93.8 (15.8)	88.8 (14.9)^b^	−4.21	<.001	−5.53	<.001	NA	NA
Full Scale IQ prorated	113.8 (12.9)	90.4 (12.0)	83.7 (12.9)^b^	−6.03	<.001	−8.10	<.001	NA	NA

Abbreviations: BMI, body mass index (calculated as weight in kilograms divided by height in meters squared); FAE, fetal alcohol effects; FAS, fetal alcohol syndrome; IQ, intelligence quotient; lm, linear model; MRI, magnetic resonance imaging; NA, not applicable.

^a^ Control vs FAE: χ^2^ = 2.115; *P* = .15; control vs FAS: χ^2^ = 7.152; *P* = .008; FAE vs FAS: χ^2^ = 1.412; *P* = .24.

^b^ Missing data for 1 participant.

### Cross-Sectional Comparison of Returners and Nonreturners

The returning participants were largely representative of the nonreturning groups, as revealed by *lm* analyses of data from the initial MRI session to test for group differences and group-by-variable interactions in participant regional brain volumes (eFigures 1-3 and eTables 1-3 in Supplement 1) and descriptors (eFigure 4 in Supplement 1), ie, age, race, educational level, handedness, body mass index, and intelligence quotients (IQ; Prorated Full Scale, Prorated Verbal, and Performance from the Wechsler Adult Intelligence Scale–Revised).^41^ Specifically, the returners did not differ significantly from the nonreturners in any regional brain volume, body mass index, or handedness. Educational level was higher in the returners than nonreturners of the FAE group, and overall, returners had higher prorated verbal IQ, performance IQ, and prorated full scale IQ scores than nonreturners, but no diagnostic group-by-returner status interactions were detected (eTable 3 in Supplement 1). The only demographic variable showing a group difference was race, such that only 1 of 5 African American individuals with FAE and 1 of 9 African American control participants returned, whereas both African American participants in the FAS group returned.

### Longitudinal Comparison

Longitudinal comparison of volumes uncorrected for ICV enabled within-participant analysis of changes related to age or sex. Volumes expressed as percentage of ICV provided a metric for between-group comparison for seeking potential group differences in trajectories of change with aging and with sex as possible moderators. Despite observing the usual phenomenon that women had smaller ICV than men, none of the tests seeking diagnosis-by-sex effects were significant, whether based on raw or ICV-adjusted volumes. Consequently, sex was eliminated from the *lmer* analyses.

#### Longitudinal Comparison of Volumes Uncorrected for ICV

**Figure 2.**
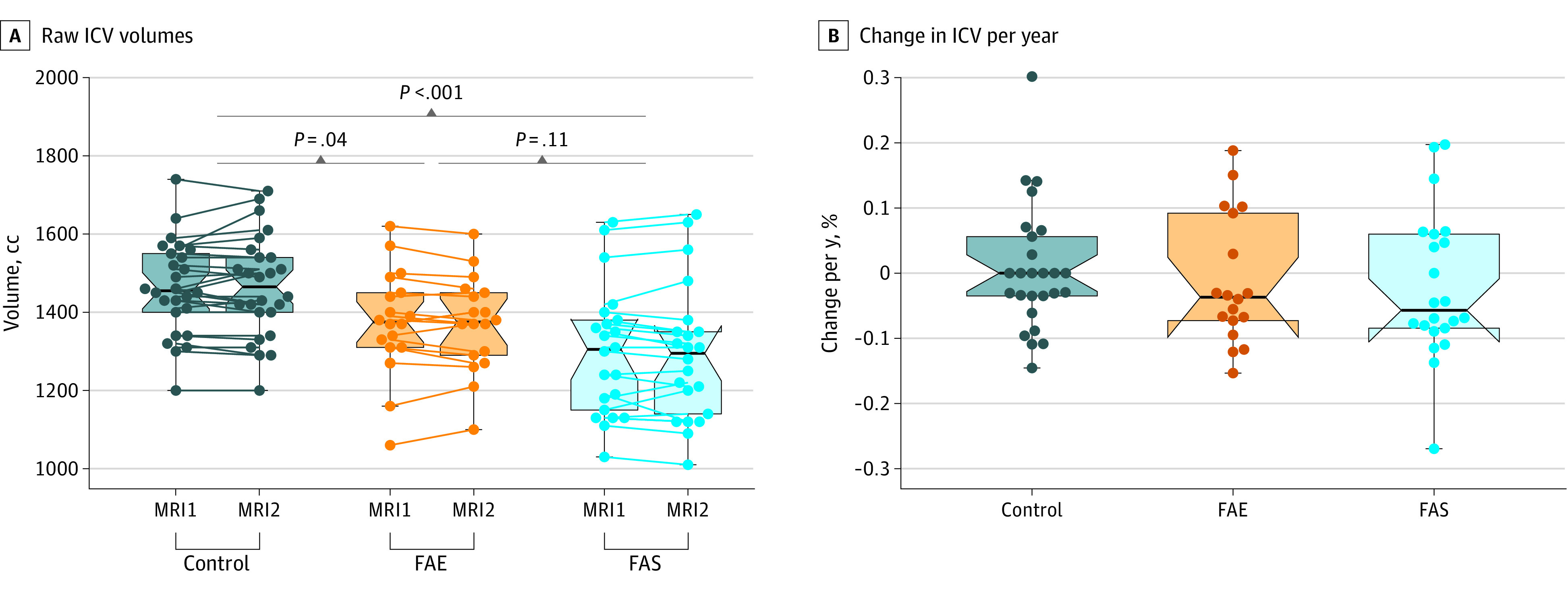
Raw Intracranial Volumes (ICV) and Percentage Change of Each Participant in Each Group Participants underwent magnetic resonance imaging (MRI) at age 13 to 37 (MRI1) and again approximately 20 years later (MRI2). The brackets at the top with *t* values and *P* values indicate the statistical results of group differences. The change in ICV of the fetal alcohol effects (FAE) and fetal alcohol syndrome (FAS) groups was not significant. Points represent individual data points; lines, change from MRI1 to MRI2; black lines, medians; boxes, IQRs; whiskers, ranges.

**Figure 3.**
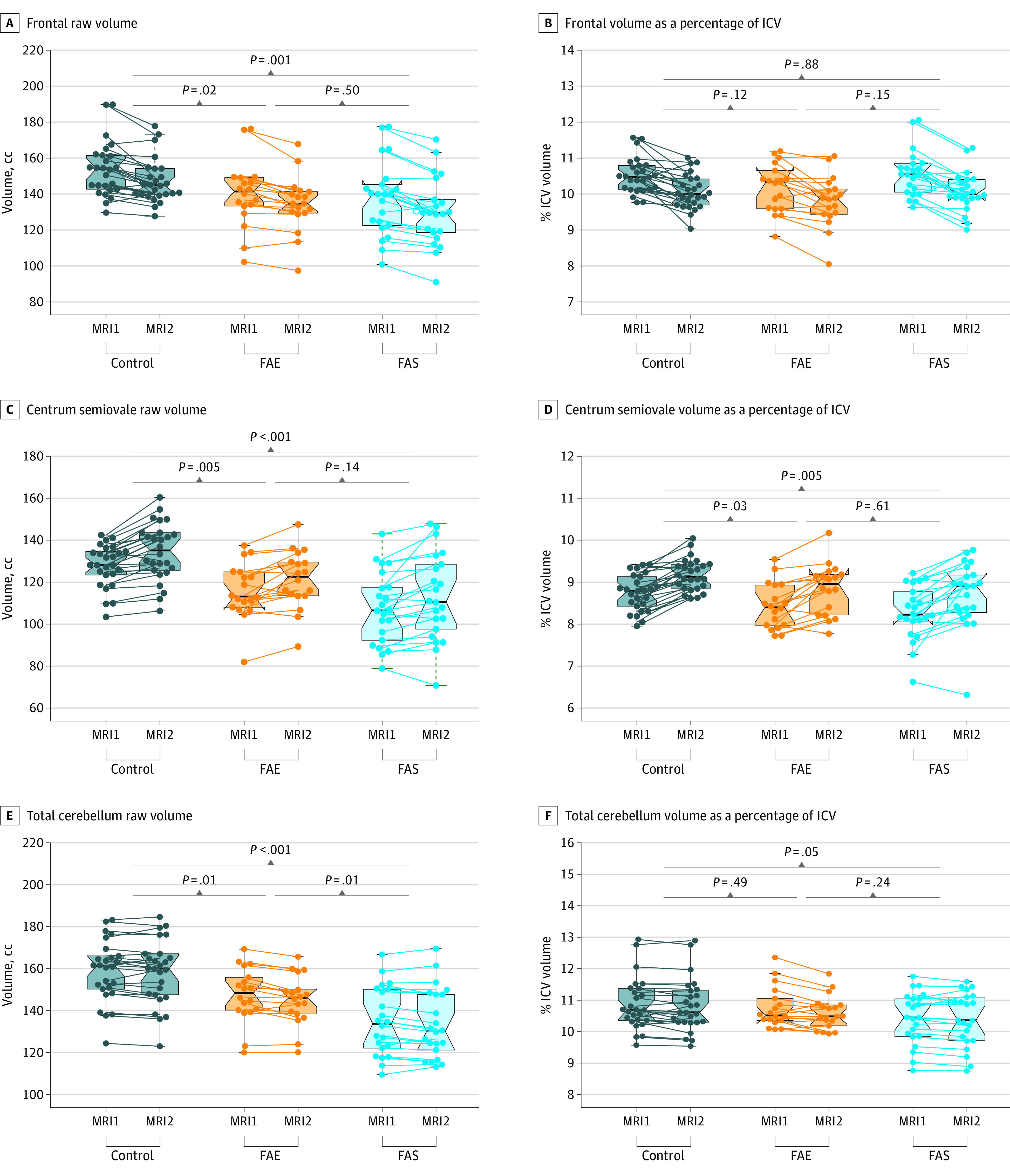
Examples of Volumetric Analysis by Group for Frontal Cortex, Centrum Semiovale White Matter, and Cerebellum Participants underwent magnetic resonance imaging (MRI) at age 13 to 37 (MRI1) and again approximately 20 years later (MRI2). The brackets at the top with *t* values and *P* values indicate the statistical results of group comparisons. FAE indicates fetal alcohol effects; FAS, fetal alcohol syndrome; ICV, intracranial volume. Points represent individual data points; lines, change from MRI1 to MRI2; black lines, medians; boxes, IQRs; whiskers, ranges.

The longitudinal analysis of ICV revealed a stepwise group volume difference (control > FAE > FAS) that was consistent over time, ie, without a significant group-by-age interaction (eg, mean [SD] ICV: control, 1462.3 [119.3] cc at MR1 and 1465.4 [129.4] cc at MRI2; FAE, 1375.6 [134.1] cc at MRI1 and 1371.7 [120.3] cc at MRI2; FAS, 1297.3 [163.0] cc at MRI1 and 1292.7 [172.1] cc at MRI2) (Figure 2 and Table 2). The overall pattern of gray matter volume change across all groups indicated significant declines in each region measured. Native (ie, raw and uncorrected for ICV) volumes of the frontal and occipital cortices showed the same significant, stepwise pattern as the ICV (eg, mean [SD] frontal lobe volume: control, 153.76 [14.97] cc at MRI1 and 147.15 cc [12.23] at MRI2; FAE, 140.07 [18.50] cc at MRI1 and 134.49 [15.49] cc at MRI2; FAS, 137.37 [20.62] cc at MRI1 and 130.18 [18.79] cc at MRI2). Volumes of the temporal, parietal, insular, and cingulate cortices also exhibited stepped differences but were significant only for the FAS group (eg, mean [SD] corpus callosum volume: control 15.41 [1.70] cc at MRI1 and 16.28 [1.90] cc at MRI2; FAE, 13.22 [1.95] cc at MRI1 and 14.00 [2.05] cc at MRI2; FAS, 12.72 [3.34] cc at MRI1 and 13.42 [3.42] cc at MRI2) (Figure 3; eFigure 5 in Supplement 1).

**Table 2.** Simple and Interaction Effects of Diagnosis and Age Indicating Acceleration of Age Outcomes^a^

Region	Diagnostic differences						FAE vs FAS		Interactions of diagnosis by age			
	Overall		FAE		FAS		*t*	*P* value	FAE		FAS	
	*t*	*P* value	*t*	*P* value	*t*	*P* value			*t*	*P* value	*t*	*P* value
**Raw MRI volumes uncorrected for ICV**												
Cortical gray matter												
ICV	−1.98	.05	−2.00	.05^b^	−3.85	<.001^c^	−1.49	.15	−0.66	.51	−0.80	.43
Frontal	−9.36	<.001^d^	−2.46	.02	−3.44	.001^c^	−0.68	.50	0.58	.56	−0.38	.70
Temporal	−17.62	<.001^d^	−0.78	.44	−2.67	.01^c^	−1.54	.13	−0.03	.98	0.12	.91
Parietal	−18.25	<.001^d^	−1.58	.12	−4.25	<.001^c^	−2.15	.04^c^	−0.27	.79	0.65	.52
Occipital	−9.94	<.001^d^	−2.05	.045^b^	−3.47	<.001^c^	−1.13	.27	−0.46	.65	−0.68	.50
Insula	−3.85	<.001^d^	−1.81	.07	−2.06	.04^c^	−0.10	.92	0.73	.47	0.45	.65
Cingulate	−10.58	<.001^d^	−1.55	.13	−2.40	.02^c^	−0.68	.50	−0.31	.76	−0.45	.66
Cortical white matter												
Corpus callosum	11.67	<.001^e^	−3.07	.003^b^	−3.91	<.001^c^	−0.53	.60	−0.43	.67	−1.16	.25
Centrum semiovale	7.81	<.001^e^	−2.90	.005^b^	−4.90	<.001^c^	−1.51	.14	−0.61	.54	−0.51	.61
Pons	13.14	<.001^e^	−2.55	.01^b^	−5.30	<.001^c^	−2.63	.01^c^	−2.56	.01^a^	−2.69	.009^c^
Cerebellum												
Total volume	−3.57	<.001^d^	−2.68	.01^b^	−5.70	<.001^c^	−2.66	.01^c^	−2.35	.02^b^	−0.94	.35
Total gray matter	4.14	<.001^e^	−2.72	.008^b^	−5.72	<.001^c^	−2.60	.01^c^	−0.44	.66	0.28	.78
Total white matter	−7.25	<.001^d^	−2.04	.045^b^	−5.11	<.001^c^	−2.70	.01^c^	−1.09	.28	−0.85	.40
Vermis gray matter	3.96	<.001^e^	−2.28	.03^b^	−4.98	<.001^c^	−2.13	.04^c^	−0.99	.33	−0.73	.47
**MRI volumes as percentages corrected for ICV**												
Cortical gray matter												
Frontal	−9.09	<.001^d^	−1.56	.12	0.16	.88	1.47	.15	0.98	.33	−0.68	.50
Temporal	−21.57	<.001^d^	1.61	.11	1.46	.15	−0.22	.83	−0.07	.94	−0.65	.52
Parietal	−22.92	<.001^d^	0.47	.64	−1.25	.22	−1.59	.12	−0.37	.71	0.21	.83
Occipital	−13.00	<.001^d^	−0.91	.37	−0.44	.66	0.45	.65	−0.49	.62	−1.13	.26
Insula	−4.39	<.001^d^	−0.24	.81	1.73	.09	1.62	.11	1.10	.27	0.66	.51
Cingulate	−13.90	<.001^d^	−0.15	.88	1.02	.31	1.01	.32	−0.21	.84	−1.08	.28
Cortical white matter												
Corpus callosum	12.07	<.001^e^	−2.58	.01^b^	−2.34	.02^c^	0.31	.76	0.26	.79	−0.10	.92
Centrum semiovale	9.74	<.001^e^	−2.17	.03^b^	−2.95	.005^c^	−0.51	.61	−0.17	.86	0.16	.88
Pons	13.39	<.001^e^	−1.24	.22	−2.81	.007^c^	−1.51	.14	−1.75	.08	−1.21	.23
Cerebellum												
Total volume	−3.51	<.001^d^	−0.70	.49	−1.97	.05	−1.20	.24	−1.85	.07	0.14	.89
Total gray matter	3.79	<.001^e^	−1.02	.31	−2.53	.01^c^	−1.30	.20	−0.38	.70	0.58	.57
Total white matter	−8.56	.001^d^	0.11	.92	−0.94	.35	−0.92	.36	−0.95	.35	−0.92	.36
Vermis gray matter	3.90	<.001^e^	−1.27	.21	−3.00	.004^c^	−1.41	.17	−0.75	.46	−0.11	.91

Abbreviations: FAE, fetal alcohol exposure; FAS, fetal alcohol syndrome; ICV, intracranial volume; MRI, magnetic resonance imaging.

^a^ Analyses conducted using linear mixed effects regression models.

^b^ Significant difference between FAE vs controls.

^c^ Significant difference between FAS vs controls.

^d^ Decreased volume with age across the 3 groups.

^e^ Increased volume with age across the 3 groups.

Whereas gray matter volumes declined with aging, white matter volumes enlarged significantly in all groups. Despite growth, the centrum semiovale, corpus callosum, and pons each displayed graded volume deficits associated with the FASD groups (Figure 3 and Table 2; eFigure 6 in Supplement 1). Only the pons showed a group-by-age interaction, where the rate of increase in pontine volume was higher in the controls than in the FAE group and the FAS group, and there was a stepwise association, with the volume of the FAS group smaller than that of the FAE group.

In contrast with cortical volumes, the overall cerebellar pattern was for gray matter to enlarge and white matter to decrease. Without adjustment for ICV, the 4 cerebellar regions examined (total tissue + cerebral spinal fluid, total gray matter, total white matter, and vermis gray matter) were marked by significant stepwise volume differences associated with diagnostic severity, without significant diagnosis by age interactions (Figure 3 and Table 2; eFigure 7 in Supplement 1). Only the FAE group showed faster decline of the total cerebellar volume than controls (mean [SD] cerebellum gray matter: control, 77.37 [9.61] cc at MRI1 and 79.24 [7.91] cc at MRI2; FAE, 70.65 [8.01] cc at MRI1 and 72.36 [7.09] cc at MRI2; FAS, 63.45 [9.33] cc at MRI1 and 65.69 [8.74] cc at MRI2).

#### Longitudinal Comparison of Volumes Corrected for ICV

ICV adjustment did not alter the pattern observed with the native cortical data, where gray matter decreased while white matter increased with age; however, ICV adjustment eliminated the group differences observed with raw volumes in all 6 cortical gray matter regions (mean [SD] frontal gray matter volume percentage: control, 10.51% [0.49%] at MRI1 and 10.06% [0.48%] at MRI2; FAE, 10.17% [0.68%] at MRI1 and 9.80% [0.71%] at MRI2; FAS, 10.57% [0.64%] at MRI1 and 10.07% [0.55%] at MRI2). At the initial MRI, the ICV of the FAE group was 94.0% and the FAS group was 89.1% of controls; at follow-up, the FAE group was 93.6% and the FAS group was 88.5% of the control group. Although all ICV-adjusted cortical volumes declined with age, the decline was not greater in the alcohol-exposed groups compared with the control group (Figure 3; eFigure 5 in Supplement 1).

In contrast with gray matter, ICV-adjusted volumes of the centrum semiovale and corpus callosum were significantly smaller in both the FAE and FAS groups than in the control group (eg, mean [SD] centrum semiovale volume percentage: control, 8.74% [0.43%] at MRI1 and 9.18% [0.38%] at MRI2; FAE, 8.42% [0.54%] at MRI1 and 8.84% [0.60%] at MRI2; FAS, 8.27% [0.64%] at MRI1 and 8.71% [0.76%] at MRI2), albeit attenuated compared with the raw volumes. The same pattern emerged for the pons although only the difference between the FAS and control groups was significant (eg, mean [SD] pons volume percentage: control, 0.57% [0.07%] at MRI1 and 0.61% [0.07%] at MRI2; FAE, 0.55% [0.06%] at MRI1 and 0.58% [0.06%] at MRI2; FAS, 0.52% [0.05%] at MRI1 and 0.55% [0.06%] at MRI2). None of the white matter volumes showed significant interaction with age in either the FAE or FAS groups compared with the controls (Figure 3 and Table 2; eFigure 6 in Supplement 1).

With ICV correction, the overall pattern of increasing cerebellar gray matter and decreasing white matter endured. Only cerebellar total gray matter and the vermis were smaller in the FAS group than the control group (eg, mean [SD] cerebellum gray matter volume percentage: control, 5.30% [0.56%] at MRI1 and 5.42% [0.47%] at MRI2; FAE, 5.15% [0.54%] at MRI1 and 5.28% [0.37%] at MRI2; FAS, 4.91% [0.54%] at MRI1 and 5.11% [0.54%] at MRI2). The diagnosis-by-age interaction was not significant in any cerebellar volume or for either the FAE or FAS group after adjustment for ICV Figure 3 and Table 2; eFigure 7 in Supplement 1.

## Discussion

This cohort study, based on longitudinal MRI data acquired in people with and without FASD over an approximately 20-year interval, identified the same stepped volume differences at MRI1 and MRI2: the volumes of the control group were larger than those of the FAE group, which were in turn larger than those of the FAS group in ICV and cerebellum and cortical regions. All groups showed patterns of volume changes as a function of age that were within reference ranges, where cortical, but not cerebellar gray matter, volumes declined over time, while cortical white matter, but not cerebellar, volumes expanded over the 20-year interval. There was not evidence of premature aging, at least as determined by brain volumetrics in the FASD groups compared with controls. The pons volumes uncorrected for ICV showed a group-by-age interaction, with the rate of volume increasing less in the FAE and FAS groups than in controls, although these diagnostic differences did not endure statistically when corrected for ICV. We are unaware of any previous reports examining the pons in cases of FASD, although an analysis of 174 autopsy cases of FASD or PAE^42^ reported 12 individuals with hypoxic-related apoptotic neurons in the pons and 1 individual with a “distorted” pons. Animal data also suggest that the pontine nucleus may be sensitive to PAE.^43^

In the larger sample of 174 participants, we previously reported ICV differences from the controls of 8% in the FAE group and 12.8% in the FAS group, which is similar to other reports.^44,45,46^ With the 66 participants remaining in the study at MRI2, similar differences were observed.

The mean age at MRI2 was 41 to 43 years, which may be too early in adulthood to discern nonlinear volumetric changes related to age. In longitudinal studies of aging, notably accelerating volume declines typically emerge at approximately age 50 years.^30,32^ There is a critical need to extend the longitudinal assessment of this cohort into older ages when clearer signs of accelerated aging might manifest morphologically to track whether the FASD population is at heightened risk for premature or exacerbated dementia or other disorders of aging. A volunteer nonprobability-type sampling survey of 541 adults who self-identified as having an FASD indicated 4 individuals with dementia among individuals aged 59 years or younger.^47^ This prevalence is significantly higher than would be expected in unaffected groups.

As expected, this study demonstrated stepwise associations in several measures, with the FAS group showing the greatest difference compared with the controls and with the FAE group being generally intermediate. Several MRI studies^48,49^ have previously reported such stepwise associations in children and adolescents in various brain regions, including ICV. These stepped deficits support a spectrum of outcomes following PAE, with the largest changes, at least as related to brain alterations, in individuals with the most dysmorphic features. This pattern of outcomes also supports the diagnostic distinctions made between FAS and other nondysmorphic disorders on the spectrum.^50,51^ However, the consequences of FASD go beyond the dysmorphic face and graded brain changes; often, the most affected individuals in terms of secondary disabilities, such as social or emotional, legal, substance use, academic, and employment issues, are nondysmorphic.^52^

FASD is a life-long condition, although diagnostic issues hamper its recognition in adulthood. Facial features change with age, and PAE information may be unavailable. Early life experiences can be difficult to identify reliably and to untangle statistically. Nonetheless, individuals with FASD continue to experience significant challenges into adulthood. In 2008, Spohr and Steinhausen^53^ reported that only 30% of a group of individuals diagnosed in early childhood with FASD and provided with protective factors lived independently, and 86% had no long-term employment history. A 2022 study by Coles et al^54^ reported higher levels of mental health disorders, particularly depression, in midlife adults with FASD, which they concluded may be mediated by a vulnerability to early life stressors resulting from PAE. The results of our study showing persistent volumetric changes into middle age may be relevant in terms of assisting with identifying FASD in adulthood, where the number of undiagnosed cases may be in the millions.^55,56^ Although not always feasible, neuroimaging as ancillary information to FASD diagnosis might prove useful in significant forensic cases.^57^ It is notable that the macrostructural differences marking FASD endured over 40 years, considering that the sample scanned in mid-life may be in better condition than those lost to follow-up. While there were few demographic differences between the rescanned group and the attritted group, the sense was that those who participated in this follow-up study represented individuals with higher functioning and the most support. This was sustained by the observation that MRI2 participants had higher IQ scores at MRI1 than nonreturners.

### Limitations

This study has some limitations. We used legacy MRI1 data acquired 2 decades ago and MRI2 data acquired 20 years later using higher-resolution parameters. Current analytic approaches enabled data harmonization to pursue questions about within-participant change over time yet precluded lobular time-linked comparison of the cerebellum. Despite the defunct descriptive and physiognomic criteria used for the initial diagnosis of FAE, volumetric analysis was successful in identifying enduring diagnostically linked, stepwise volume deficits in cortical and cerebellar tissue. Furthermore, a loss to follow-up resulted in relatively small samples, although the findings were adequately robust to detect stepped associations and expected age differences. There is a clear need for further follow-up to extend to age ranges to when brain structural volume declines typically become apparent and may reveal accelerated declines in the FASD groups.^30,32,58^ Additional measurements would also enable modeling aging trajectories, which may differ across the 3 study groups.

## Conclusions

To our knowledge, this cohort study presents longitudinal brain findings over 20 years on the oldest studied group of individuals diagnosed with FASD. The results indicate enduring stepwise changes with little evidence of accelerated brain aging or reversal of regional volume deficits. These data support an enduring spectrum within this age range based on the original severity of dysmorphia and a diagnostic distinction between FAS and FAE. Despite prenatal alcohol-induced brain structural deficits with clinically observable behavioral consequences, our findings do not suggest that FASD is a progressive disorder by middle age but one for which continued habilitative efforts are warranted.

## Notes

Supplement 1. **eTable 1.** Comparison of Returners vs Nonreturners **eTable 2.** Mean, SD, SE, and N for Nonreturners and Returners by Group **eTable 3.** Tests for Control-by-FAE or FAS Interaction of Returners vs Nonreturners **eFigure 1.** Intracranial Volumes (ICV) for Returning vs Nonreturning Participants **eFigure 2.** Volumes of Cortical Regions for Returning vs Nonreturning Participants **eFigure 3.** White Matter and Ventricular Measures for Returning vs Nonreturning Participants **eFigure 4.** Demographic Data for Returning vs Nonreturning Participants **eFigure 5.** Raw and ICV-Corrected Volumes of Cortical Gray Matter Regions at Each MRI Session **eFigure 6.** Raw and ICV-Corrected Volumes of White Matter Regions at Each MRI Session **eFigure 7.** Raw and ICV-Corrected Volumes of Cerebellar Regions at Each MRI Session

Supplement 2.
Data Sharing Statement

## References

[1] Popova S, Charness ME, Burd L, . Fetal alcohol spectrum disorders. Nat Rev Dis Primers. 2023;9(1):11. doi:10.1038/s41572-023-00420-x 36823161

[2] Wozniak JR, Riley EP, Charness ME. Clinical presentation, diagnosis, and management of fetal alcohol spectrum disorder. Lancet Neurol. 2019;18(8):760-770. doi:10.1016/S1474-4422(19)30150-4 31160204 PMC6995665

[3] Bookstein FL, Sampson PD, Connor PD, Streissguth AP. Midline corpus callosum is a neuroanatomical focus of fetal alcohol damage. Anat Rec. 2002;269(3):162-174. doi:10.1002/ar.10110 12124903

[4] Boronat S, Sánchez-Montañez A, Gómez-Barros N, . Correlation between morphological MRI findings and specific diagnostic categories in fetal alcohol spectrum disorders. Eur J Med Genet. 2017;60(1):65-71. doi:10.1016/j.ejmg.2016.09.003 27620364

[5] Nguyen VT, Chong S, Tieng QM, Mardon K, Galloway GJ, Kurniawan ND. Radiological studies of fetal alcohol spectrum disorders in humans and animal models: an updated comprehensive review. Magn Reson Imaging. 2017;43:10-26. doi:10.1016/j.mri.2017.06.012 28645698

[6] Roebuck TM, Mattson SN, Riley EP. A review of the neuroanatomical findings in children with fetal alcohol syndrome or prenatal exposure to alcohol. Alcohol Clin Exp Res. 1998;22(2):339-344. doi:10.1111/j.1530-0277.1998.tb03658.x 9581638

[7] Glass L, Ware AL, Mattson SN. Neurobehavioral, neurologic, and neuroimaging characteristics of fetal alcohol spectrum disorders. Handb Clin Neurol. 2014;125:435-462. doi:10.1016/B978-0-444-62619-6.00025-2 25307589

[8] Riley EP, Infante MA, Warren KR. Fetal alcohol spectrum disorders: an overview. Neuropsychol Rev. 2011;21(2):73-80. doi:10.1007/s11065-011-9166-x 21499711 PMC3779274

[9] Rössig C, Wässer S, Oppermann P. Audiologic manifestations in fetal alcohol syndrome assessed by brainstem auditory-evoked potentials. Neuropediatrics. 1994;25(5):245-249. doi:10.1055/s-2008-1073029 7885533

[10] Jirikowic TL, Thorne JC, McLaughlin SA, Waddington T, Lee AKC, Astley Hemingway SJ. Prevalence and patterns of sensory processing behaviors in a large clinical sample of children with prenatal alcohol exposure. Res Dev Disabil. 2020;100:103617. doi:10.1016/j.ridd.2020.103617 32203885

[11] Coffman BA, Candelaria-Cook FT, Stephen JM. Unisensory and multisensory responses in fetal alcohol spectrum disorders (FASD): effects of spatial congruence. Neuroscience. 2020;430:34-46. doi:10.1016/j.neuroscience.2020.01.013 31982473 PMC7098157

[12] McLaughlin SA, Thorne JC, Jirikowic T, Waddington T, Lee AKC, Astley Hemingway SJ. Listening difficulties in children with fetal alcohol spectrum disorders: more than a problem of audibility. J Speech Lang Hear Res. 2019;62(5):1532-1548. doi:10.1044/2018_JSLHR-H-18-0359 31039324

[13] Paolozza A, Rasmussen C, Pei J, . Working memory and visuospatial deficits correlate with oculomotor control in children with fetal alcohol spectrum disorder. Behav Brain Res. 2014;263:70-79. doi:10.1016/j.bbr.2014.01.024 24486257

[14] Domellöf E, Fagard J, Jacquet AY, Rönnqvist L. Goal-directed arm movements in children with fetal alcohol syndrome: a kinematic approach. Eur J Neurol. 2011;18(2):312-320. doi:10.1111/j.1468-1331.2010.03142.x 20629717

[15] Gautam P, Nuñez SC, Narr KL, . Developmental trajectories for visuo-spatial attention are altered by prenatal alcohol exposure: a longitudinal FMRI study. Cereb Cortex. 2015;25(12):4761-4771. doi:10.1093/cercor/bhu162 25092900 PMC4635917

[16] Gross LA, Moore EM, Wozniak JR, ; CIFASD. Neural correlates of verbal memory in youth with heavy prenatal alcohol exposure. Brain Imaging Behav. 2018;12(3):806-822. doi:10.1007/s11682-017-9739-2 28656347 PMC5745322

[17] Willoughby KA, Sheard ED, Nash K, Rovet J. Effects of prenatal alcohol exposure on hippocampal volume, verbal learning, and verbal and spatial recall in late childhood. J Int Neuropsychol Soc. 2008;14(6):1022-1033. doi:10.1017/S1355617708081368 18954482

[18] Inkelis SM, Moore EM, Bischoff-Grethe A, Riley EP. Neurodevelopment in adolescents and adults with fetal alcohol spectrum disorders (FASD): a magnetic resonance region of interest analysis. Brain Res. 2020;1732(146654):146654. doi:10.1016/j.brainres.2020.146654 31930998 PMC7067519

[19] Sullivan EV, Moore EM, Lane B, Pohl KM, Riley EP, Pfefferbaum A. Graded cerebellar lobular volume deficits in adolescents and young adults with fetal alcohol spectrum disorders (FASD). Cereb Cortex. 2020;30(9):4729-4746. doi:10.1093/cercor/bhaa020 32133485 PMC7391273

[20] Moore EM, Riley EP. What happens when children with fetal alcohol spectrum disorders become adults? Curr Dev Disord Rep. 2015;2(3):219-227. doi:10.1007/s40474-015-0053-7 26543794 PMC4629517

[21] Ma X, Coles CD, Lynch ME, . Evaluation of corpus callosum anisotropy in young adults with fetal alcohol syndrome according to diffusion tensor imaging. Alcohol Clin Exp Res. 2005;29(7):1214-1222. doi:10.1097/01.ALC.0000171934.22755.6D 16046877

[22] Li L, Coles CD, Lynch ME, Hu X. Voxelwise and skeleton-based region of interest analysis of fetal alcohol syndrome and fetal alcohol spectrum disorders in young adults. Hum Brain Mapp. 2009;30(10):3265-3274. doi:10.1002/hbm.20747 19278010 PMC2748153

[23] Chen X, Coles CD, Lynch ME, Hu X. Understanding specific effects of prenatal alcohol exposure on brain structure in young adults. Hum Brain Mapp. 2012;33(7):1663-1676. doi:10.1002/hbm.21313 21692145 PMC3755753

[24] Moore EM, Xia Y. Neurodevelopmental trajectories following prenatal alcohol exposure. Front Hum Neurosci. 2022;15:695855. doi:10.3389/fnhum.2021.695855 35058760 PMC8763806

[25] Lebel C, Mattson SN, Riley EP, . A longitudinal study of the long-term consequences of drinking during pregnancy: heavy in utero alcohol exposure disrupts the normal processes of brain development. J Neurosci. 2012;32(44):15243-15251. doi:10.1523/JNEUROSCI.1161-12.2012 23115162 PMC3515671

[26] Treit S, Zhou D, Lebel C, Rasmussen C, Andrew G, Beaulieu C. Longitudinal MRI reveals impaired cortical thinning in children and adolescents prenatally exposed to alcohol. Hum Brain Mapp. 2014;35(9):4892-4903. doi:10.1002/hbm.22520 24700453 PMC6869356

[27] Hendrickson TJ, Mueller BA, Sowell ER, . Two-year cortical trajectories are abnormal in children and adolescents with prenatal alcohol exposure. Dev Cogn Neurosci. 2018;30:123-133. doi:10.1016/j.dcn.2018.02.008 29486453 PMC5949095

[28] Mason D, Ronald A, Ambler A, . Autistic traits are associated with faster pace of aging: evidence from the Dunedin study at age 45. Autism Res. 2021;14(8):1684-1694. doi:10.1002/aur.2534 34042279 PMC8328948

[29] Horvath S, Garagnani P, Bacalini MG, . Accelerated epigenetic aging in Down syndrome. Aging Cell. 2015;14(3):491-495. doi:10.1111/acel.12325 25678027 PMC4406678

[30] Bethlehem RAI, Seidlitz J, White SR, ; 3R-BRAIN; AIBL; Alzheimer’s Disease Neuroimaging Initiative; Alzheimer’s Disease Repository Without Borders Investigators; CALM Team; Cam-CAN; CCNP; COBRE; cVEDA; ENIGMA Developmental Brain Age Working Group; Developing Human Connectome Project; FinnBrain; Harvard Aging Brain Study; IMAGEN; KNE96; Mayo Clinic Study of Aging; NSPN; POND; PREVENT-AD Research Group; VETSA. Brain charts for the human lifespan. Nature. 2022;604(7906):525-533. doi:10.1038/s41586-022-04554-y 35388223 PMC9021021

[31] Pfefferbaum A, Kwon D, Brumback T, . Altered brain developmental trajectories in adolescents after initiating drinking. Am J Psychiatry. 2018;175(4):370-380. doi:10.1176/appi.ajp.2017.17040469 29084454 PMC6504929

[32] Pfefferbaum A, Rohlfing T, Rosenbloom MJ, Chu W, Colrain IM, Sullivan EV. Variation in longitudinal trajectories of regional brain volumes of healthy men and women (ages 10 to 85 years) measured with atlas-based parcellation of MRI. Neuroimage. 2013;65:176-193. doi:10.1016/j.neuroimage.2012.10.008 23063452 PMC3516371

[33] Riley EP, Pohl KM, Grethe A, Stoner SA, Sullivan EV, Pfefferbaum A. Graded regional cerebellar volume deficits in adolescents and adults with fetal alcohol effect (FAE) and fetal alcohol syndrome (FAS). Alcohol. 2023;109:91-92. doi:10.1016/j.alcohol.2023.03.101

[34] Bookstein FL, Sampson PD, Streissguth AP, Connor PD. Geometric morphometrics of corpus callosum and subcortical structures in the fetal-alcohol-affected brain. Teratology. 2001;64(1):4-32. doi:10.1002/tera.1044 11410908

[35] Bookstein FL, Streissguth AP, Sampson PD, Connor PD, Barr HM. Corpus callosum shape and neuropsychological deficits in adult males with heavy fetal alcohol exposure. Neuroimage. 2002;15(1):233-251. doi:10.1006/nimg.2001.0977 11771992

[36] Streissguth AP, Aase JM, Clarren SK, Randels SP, LaDue RA, Smith DF. Fetal alcohol syndrome in adolescents and adults. JAMA. 1991;265(15):1961-1967. doi:10.1001/jama.1991.03460150065025 2008025

[37] Rohlfing T, Zahr NM, Sullivan EV, Pfefferbaum A. The SRI24 multichannel atlas of normal adult human brain structure. Hum Brain Mapp. 2010;31(5):798-819. doi:10.1002/hbm.20906 20017133 PMC2915788

[38] Avants BB, Epstein CL, Grossman M, Gee JC. Symmetric diffeomorphic image registration with cross-correlation: evaluating automated labeling of elderly and neurodegenerative brain. Med Image Anal. 2008;12(1):26-41. doi:10.1016/j.media.2007.06.004 17659998 PMC2276735

[39] Avants BB, Tustison NJ, Song G, Cook PA, Klein A, Gee JC. A reproducible evaluation of ANTs similarity metric performance in brain image registration. Neuroimage. 2011;54(3):2033-2044. doi:10.1016/j.neuroimage.2010.09.025 20851191 PMC3065962

[40] Diedrichsen J. A spatially unbiased atlas template of the human cerebellum. Neuroimage. 2006;33(1):127-138. doi:10.1016/j.neuroimage.2006.05.056 16904911

[41] Wechsler D. Wechsler Adult Intelligence Scale–Revised. Psychological Corporation; 1981.

[42] Jarmasz JS, Basalah DA, Chudley AE, Del Bigio MR. Human brain abnormalities associated with prenatal alcohol exposure and fetal alcohol spectrum disorder. J Neuropathol Exp Neurol. 2017;76(9):813-833. doi:10.1093/jnen/nlx064 28859338 PMC5901082

[43] Dikranian K, Qin Y-Q, Labruyere J, Nemmers B, Olney JW. Ethanol-induced neuroapoptosis in the developing rodent cerebellum and related brain stem structures. Brain Res Dev Brain Res. 2005;155(1):1-13. doi:10.1016/j.devbrainres.2004.11.005 15763270

[44] Riley EP, Mattson SN, Sowell ER, Jernigan TL, Sobel DF, Jones KL. Abnormalities of the corpus callosum in children prenatally exposed to alcohol. Alcohol Clin Exp Res. 1995;19(5):1198-1202. doi:10.1111/j.1530-0277.1995.tb01600.x 8561290

[45] Astley SJ, Aylward EH, Olson HC, . Magnetic resonance imaging outcomes from a comprehensive magnetic resonance study of children with fetal alcohol spectrum disorders. Alcohol Clin Exp Res. 2009;33(10):1671-1689. doi:10.1111/j.1530-0277.2009.01004.x 19572986 PMC4170878

[46] Lebel C, Rasmussen C, Wyper K, . Brain diffusion abnormalities in children with fetal alcohol spectrum disorder. Alcohol Clin Exp Res. 2008;32(10):1732-1740. doi:10.1111/j.1530-0277.2008.00750.x 18671811

[47] Himmelreich M, Lutke CJ, Hargrove E. The lay of the land: Fetal alcohol spectrum disorder (FASD) as a whole-body diagnosis. In: Begun AL, Murray MM, . The Routledge Handbook of Social Work and Addictive Behaviours. Routledge; 2020:191-215. doi:10.4324/9780429203121-14

[48] Norman AL, Crocker N, Mattson SN, Riley EP. Neuroimaging and fetal alcohol spectrum disorders. Dev Disabil Res Rev. 2009;15(3):209-217. doi:10.1002/ddrr.72 19731391 PMC3442778

[49] Lebel C, Roussotte F, Sowell ER. Imaging the impact of prenatal alcohol exposure on the structure of the developing human brain. Neuropsychol Rev. 2011;21(2):102-118. doi:10.1007/s11065-011-9163-0 21369875 PMC3098972

[50] Hoyme HE, Kalberg WO, Elliott AJ, . Updated clinical guidelines for diagnosing fetal alcohol spectrum disorders. Pediatrics. 2016;138(2):e20154256. doi:10.1542/peds.2015-4256 27464676 PMC4960726

[51] Cook JL, Green CR, Lilley CM, ; Canada Fetal Alcohol Spectrum Disorder Research Network. Fetal alcohol spectrum disorder: a guideline for diagnosis across the lifespan. CMAJ. 2016;188(3):191-197. doi:10.1503/cmaj.141593 26668194 PMC4754181

[52] Petrenko CL, Tahir N, Mahoney EC, Chin NP. Prevention of secondary conditions in fetal alcohol spectrum disorders: identification of systems-level barriers. Matern Child Health J. 2014;18(6):1496-1505. doi:10.1007/s10995-013-1390-y 24178158 PMC4007413

[53] Spohr HL, Steinhausen HC. Fetal alcohol spectrum disorders and their persisting sequelae in adult life. Dtsch Arztebl Int. 2008;105(41):693-698. doi:10.3238/arztebl.2008.0693 19623288 PMC2696967

[54] Coles CD, Grant TM, Kable JA, Stoner SA, Perez A; Collaborative Initiative on Fetal Alcohol Spectrum Disorders. Prenatal alcohol exposure and mental health at midlife: a preliminary report on two longitudinal cohorts. Alcohol Clin Exp Res. 2022;46(2):232-242. doi:10.1111/acer.14761 35157325 PMC8867925

[55] Burd L, Popova S. Fetal alcohol spectrum disorders: fixing our aim to aim for the fix. Int J Environ Res Public Health. 2019;16(20):3978. doi:10.3390/ijerph16203978 31635265 PMC6843765

[56] Popova S, Dozet D, Burd L. Fetal alcohol spectrum disorder: can we change the future? Alcohol Clin Exp Res. 2020;44(4):815-819. doi:10.1111/acer.14317 32128856 PMC7217166

[57] Perlin ML, Cucolo H. ‘Take the motherless children off the street’: Fetal alcohol syndrome and the criminal justice system. NYLS Legal Studies Research Paper No. 4209258. *SSRN*. Preprint posted online September 12, 2022. doi:10.2139/ssrn.4209258

[58] Raz N, Ghisletta P, Rodrigue KM, Kennedy KM, Lindenberger U. Trajectories of brain aging in middle-aged and older adults: regional and individual differences. Neuroimage. 2010;51(2):501-511. doi:10.1016/j.neuroimage.2010.03.020 20298790 PMC2879584

