# Supplementary material for: Brain Volume in Fetal Alcohol Spectrum Disorders Over a 20-Year Span
Source: JAMA Netw Open. 2023 Nov 17;6(11):e2343618. doi: 10.1001/jamanetworkopen.2023.43618 (PMC10656646; doi:10.1001/jamanetworkopen.2023.43618)
Supplement: Supplement 1. — eTable 1. Comparison of Returners vs Nonreturners eTable 2. Mean, SD, SE, and N for Nonreturners and Returners by Group eTable 3. Tests for Control-by-FAE or FAS Interaction of Returners vs Nonreturners eFigure 1. Intracranial Volumes (ICV) for Returning vs Nonreturning Participants eFigure 2. Volumes of Cortical Regions for Returning vs Nonreturning Participants eFigure 3. White Matter and Ventricular Measures for Returning vs Nonreturning Participants eFigure 4. Demographic Data for Returning vs Nonreturning Participants eFigure 5. Raw and ICV-Corrected Volumes of Cortical Gray Matter Regions at Each MRI Session eFigure 6. Raw and ICV-Corrected Volumes of White Matter Regions at Each MRI Session eFigure 7. Raw and ICV-Corrected Volumes of Cerebellar Regions at Each MRI Session [file jamanetwopen-e2343618-s001.pdf]

Supplemental Online Content

Pfefferbaum A, Sullivan EV, Pohl KM, et al. Brain volume in fetal alcohol spectrum disorders over a 20-year span. *JAMA Netw Open*. 2023;6(11):e2343618.
doi:10.1001/jamanetworkopen.2023.43618

eTable 1. Comparison of Returners vs Nonreturners
eTable 2. Mean, SD, SE, and N for Nonreturners and Returners by Group
eTable 3. Tests for Control-by-FAE or FAS Interaction of Returners vs Nonreturners
eFigure 1. Intracranial Volumes (ICV) for Returning vs Nonreturning Participants
eFigure 2. Volumes of Cortical Regions for Returning vs Nonreturning Participants
eFigure 3. White Matter and Ventricular Measures for Returning vs Nonreturning Participants
eFigure 4. Demographic Data for Returning vs Nonreturning Participants
eFigure 5. Raw and ICV-Corrected Volumes of Cortical Gray Matter Regions at Each MRI Session
eFigure 6. Raw and ICV-Corrected Volumes of White Matter Regions at Each MRI Session
eFigure 7. Raw and ICV-Corrected Volumes of Cerebellar Regions at Each MRI Session

This supplemental material has been provided by the authors to give readers additional information about their work.

20 October 2023

© 2023 Pfefferbaum A et al. *JAMA Network Open.*

eTable 1. Comparison of Returners vs. Non-returners

	Control		FAE		FAS	
	**t**	**p**	**t**	**p**	**t**	**p**
ICV	-0.41	0.69	0.69	0.50	0.32	0.75
**Gray matter volume**						
Frontal	-0.21	0.84	0.13	0.90	0.77	0.45
Temporal	-0.47	0.64	1.32	0.19	0.40	0.69
Parietal	0.41	0.68	0.17	0.87	-0.52	0.60
Occipital	-0.76	0.45	-0.33	0.74	-0.06	0.95
Insula	-1.13	0.26	0.08	0.94	0.81	0.42
Cingulate	-1.27	0.21	0.30	0.76	0.41	0.69
**White matter volume**						
Centrum semiovale	-0.68	0.50	0.09	0.93	0.20	0.84
Corpus callosum	-0.41	0.68	-1.62	0.11	0.08	0.94
Pons	-0.90	0.37	0.25	0.80	-0.45	0.66
**Ventricular volume**						
Lateral	0.34	0.73	0.06	0.96	0.12	0.90
Third	-0.16	0.87	2.42	**0.02**	0.93	0.36
**Demographics**						
Educational level	0.63	0.53	2.36	**0.02**	1.15	0.26
BMI	-0.47	0.64	-0.53	0.60	-0.16	0.87
Handedness	0.17	0.87	1.05	0.30	1.45	0.15
VIQ prorated	2.19	**0.03**	2.59	**0.01**	1.16	0.25
PIQ	1.95	0.06	1.93	0.06	1.73	0.09
FSIQ prorated	2.27	**0.03**	2.73	**0.01**	1.60	0.12
PIQ-VIQ discrepancy	0.33	0.74	-0.46	0.65	0.90	0.37

Bonferroni correct for 18 comparisons with a=.05 (2-tailed) requires p=.0028.

Thus, none of the uncorrected p-values met correction for multiple comparisons.

© 2023 Pfefferbaum A et al. *JAMA Network Open.*

eTable 2. Mean, SD, SE, and N for Non-returners and Returners by group

	Control							
	Non-returner				Returner			
	N	Mean	SD	SE	N	Mean	SD	SE
ICV	33	1558.33	179.55	31.25	26	1541.59	123.96	24.31
**Gray matter volume**								
Frontal	33	179.75	21.10	3.67	26	178.72	15.61	3.06
Temporal	33	14.48	1.80	0.31	26	14.01	1.25	0.25
Parietal	33	23.17	3.63	0.63	26	22.04	3.01	0.59
Occipital	33	61.45	7.01	1.22	26	60.12	6.32	1.24
Insula	33	84.10	9.13	1.59	26	85.02	7.67	1.50
Cingulate	33	106.48	13.12	2.28	26	105.04	9.81	1.92
**White matter volume**								
Centrum semiovale	33	128.82	19.46	3.39	26	125.91	10.81	2.12
Corpus callosum	33	15.79	3.13	0.55	26	15.51	1.69	0.33
Pons	33	8.54	1.32	0.23	26	8.24	1.15	0.23
**Ventricular volume**								
Lateral	33	10.90	4.15	0.72	26	11.25	3.46	0.68
Third	33	1.24	0.17	0.03	26	1.23	0.18	0.04
**Demographics**								
Educational level	33	11.52	2.71	0.47	26	11.96	2.71	0.53
BMI	33	25.49	4.28	0.75	25	24.97	4.14	0.83
Handedness	33	0.03	0.17	0.03	26	0.04	0.20	0.04
VIQ prorated	33	105.33	12.33	2.15	26	112.00	10.60	2.08
PIQ	33	105.39	15.53	2.70	26	113.12	14.48	2.84
FSIQ prorated	33	105.88	13.65	2.38	26	113.81	12.93	2.54
PIQ-VIQ discrepancy	33	0.06	13.51	2.35	26	1.12	9.95	1.95
	FAE							
	Non-returner				Returner			
	N	Mean	SD	SE	N	Mean	SD	SE
ICV	40	1425.53	179.67	28.41	18	1458.35	140.17	33.04
**Gray matter volume**								
Frontal	40	164.23	23.92	3.78	18	165.10	22.76	5.36
Temporal	40	13.25	1.94	0.31	18	13.29	1.78	0.42
Parietal	40	20.83	3.74	0.59	18	21.14	3.30	0.78
Occipital	40	57.04	8.43	1.33	18	56.27	7.64	1.80
Insula	40	79.72	10.07	1.59	18	80.21	10.37	2.45
Cingulate	40	97.70	12.24	1.93	18	102.36	12.75	3.01
**White matter volume**								
Centrum semiovale	40	114.84	17.75	2.81	18	115.25	12.73	3.00
Corpus callosum	40	14.30	2.75	0.43	18	13.13	1.96	0.46
Pons	40	7.51	1.15	0.18	18	7.59	1.09	0.26
**Ventricular volume**								
Lateral	40	11.06	7.46	1.18	18	11.16	3.01	0.71
Third	40	1.13	0.18	0.03	18	1.28	0.30	0.07
**Demographics**								
Educational level	40	9.83	1.80	0.28	18	11.06	1.92	0.45
BMI	40	26.24	6.34	1.00	18	25.28	6.66	1.57
Handedness	40	0.08	0.27	0.04	18	0.17	0.38	0.09
VIQ prorated	40	79.63	13.41	2.12	18	89.06	11.45	2.70
PIQ	39	85.10	15.79	2.53	18	93.78	15.84	3.73
FSIQ prorated	39	79.77	14.33	2.29	18	90.39	11.96	2.82
PIQ-VIQ discrepancy	39	6.18	10.31	1.65	18	4.72	12.66	2.98
	FAS							
	Non-returner				Returner			
	N	Mean	SD	SE	N	Mean	SD	SE
ICV	36	1347.48	200.49	33.42	22	1363.92	174.77	37.26
**Gray matter volume**								
Frontal	36	153.92	23.05	3.84	22	158.74	23.56	5.02
Temporal	36	12.66	1.64	0.27	22	13.06	2.09	0.45
Parietal	36	20.07	3.80	0.63	22	20.47	3.32	0.71
Occipital	36	53.97	9.67	1.61	22	53.83	7.96	1.70
Insula	36	75.53	12.65	2.11	22	73.78	11.89	2.54
Cingulate	36	94.26	13.69	2.28	22	95.71	13.30	2.84
**White matter volume**								
Centrum semiovale	36	106.13	20.97	3.49	22	107.25	19.09	4.07
Corpus callosum	36	12.70	3.09	0.52	22	12.77	3.37	0.72
Pons	36	6.90	1.38	0.23	22	6.75	0.93	0.20
**Ventricular volume**								
Lateral	36	9.48	3.97	0.66	22	9.63	4.76	1.01
Third	36	1.06	0.21	0.03	22	1.11	0.25	0.05
**Demographics**								
Educational level	36	9.58	2.25	0.37	21	10.29	2.19	0.48
BMI	36	24.50	5.55	0.92	22	24.25	5.60	1.19
Handedness	36	0.17	0.38	0.06	21	0.33	0.48	0.11
VIQ prorated	36	78.28	13.03	2.17	21	82.24	11.44	2.50
PIQ	35	81.57	15.39	2.60	21	88.81	14.87	3.24
FSIQ prorated	35	77.86	13.47	2.28	21	83.71	12.91	2.82
PIQ-VIQ discrepancy	35	3.80	12.21	2.06	21	6.57	9.09	1.98

© 2023 Pfefferbaum A et al. *JAMA Network Open.*

eTable 3. Tests for control-by-FAE or FAS interaction of Returners vs. Non-returners								
	**Return vs. NonReturn**		**Return status+diagnosis**		**Return status* FAE diagnosis**		**Return status* FAS diagnosis**	
	**t**	**p**	**t**	**p**	**t**	**p**	**t**	**p**
ICV	0.67	0.51	0.35	0.72	0.74	0.46	0.51	0.61
**Gray matter volume**								
Frontal	0.76	0.45	0.44	0.66	0.22	0.82	0.71	0.48
Temporal	0.95	0.34	0.71	0.48	1.25	0.21	0.61	0.54
Parietal	0.17	0.86	-0.07	0.95	-0.11	0.92	-0.68	0.50
Occipital	-0.30	0.77	-0.61	0.54	0.18	0.86	0.39	0.70
Insula	0.24	0.81	-0.08	0.94	0.75	0.45	1.30	0.19
Cingulate	-0.03	0.98	-0.31	0.75	1.05	0.29	1.14	0.25
**White matter volume**								
Centrum semiovale	0.19	0.85	-0.20	0.85	0.48	0.63	0.60	0.55
Corpus callosum	-0.58	0.56	-0.99	0.32	-0.82	0.41	0.33	0.74
Pons	-0.24	0.81	-0.69	0.49	0.80	0.42	0.32	0.75
**Ventricular volume**								
Lateral	0.25	0.80	0.26	0.79	-0.13	0.90	-0.11	0.91
Third	1.99	0.05	1.93	0.06	1.99	**0.05**	0.81	0.42
**Demographics**								
Educational level	2.43	0.02	2.15	0.03	0.89	0.38	0.29	0.77
BMI	-0.73	0.46	-0.66	0.51	-0.21	0.84	0.13	0.89
Handedness	1.52	0.13	1.73	0.09	0.68	0.50	1.32	0.19
VIQ prorated	3.30	**0.001**	3.42	**0.001**	0.58	0.56	-0.58	0.56
PIQ	3.33	**0.001**	3.25	**0.001**	0.16	0.87	-0.08	0.93
FSIQ prorated	3.57	**0.000**	3.82	**0.000**	0.52	0.61	-0.41	0.69
PIQ-VIQ discrepancy	0.20	0.84	0.47	0.64	-0.57	0.57	0.39	0.69
Bonferroni correct for 18 comparisons with a=.05 (2-tailed) requires p=.0028.								
**Bold font**: Irrespective of diagnosis, Returners had, on average, higher IQ scores than Non-returners.								

© 2023 Pfefferbaum A et al. *JAMA Network Open.*

Group Differences between Non-returners and Returners

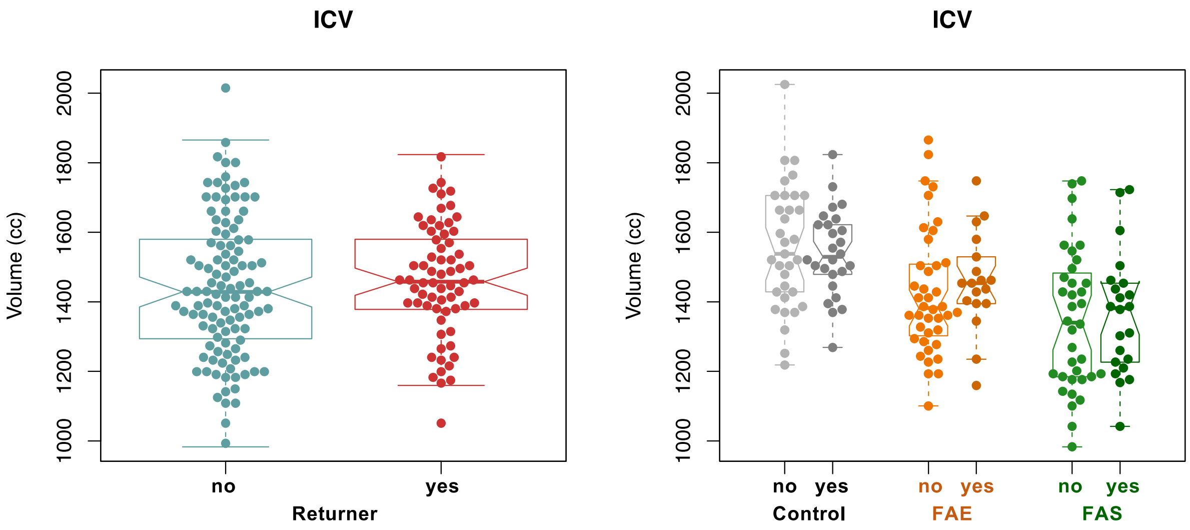

eFigure 1

© 2023 Pfefferbaum A et al. *JAMA Network Open.*

Group Differences between Non-returners and Returners

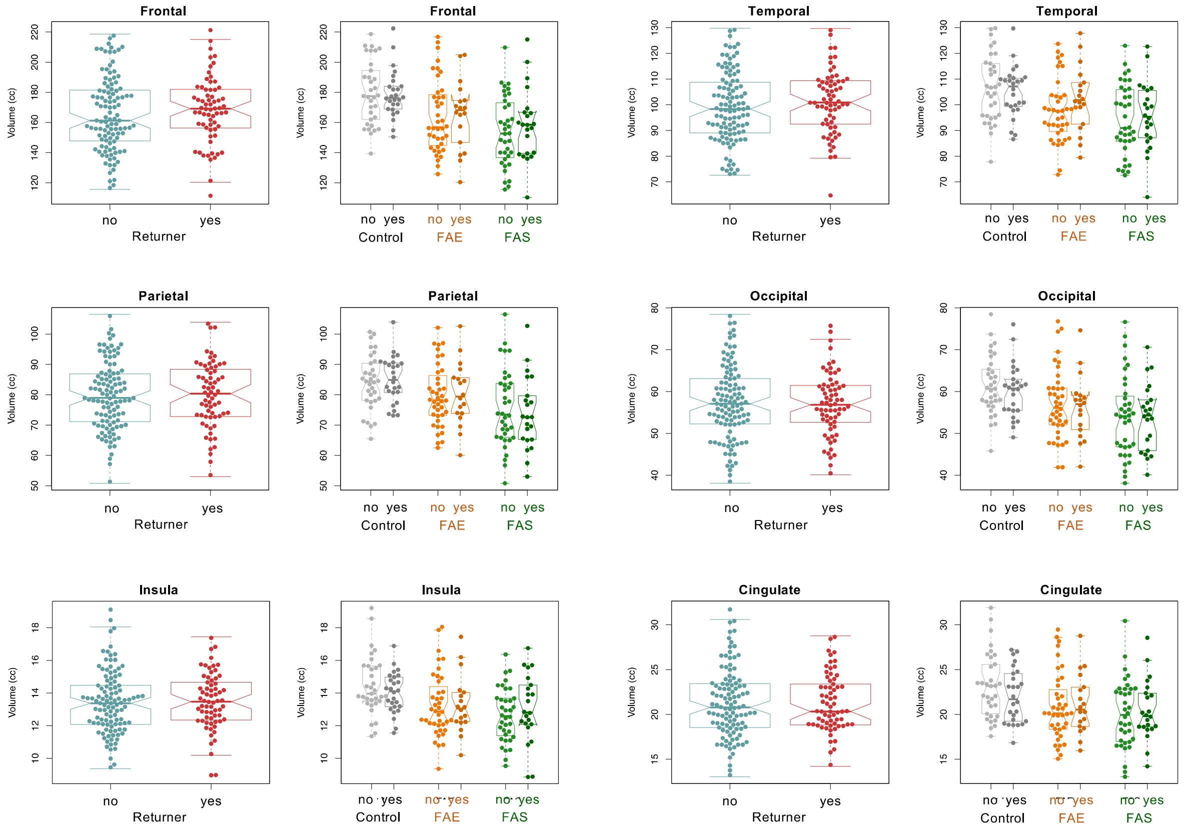

eFigure 2

© 2023 Pfefferbaum A et al. *JAMA Network Open.*

Group Differences between Non-returners and Returners

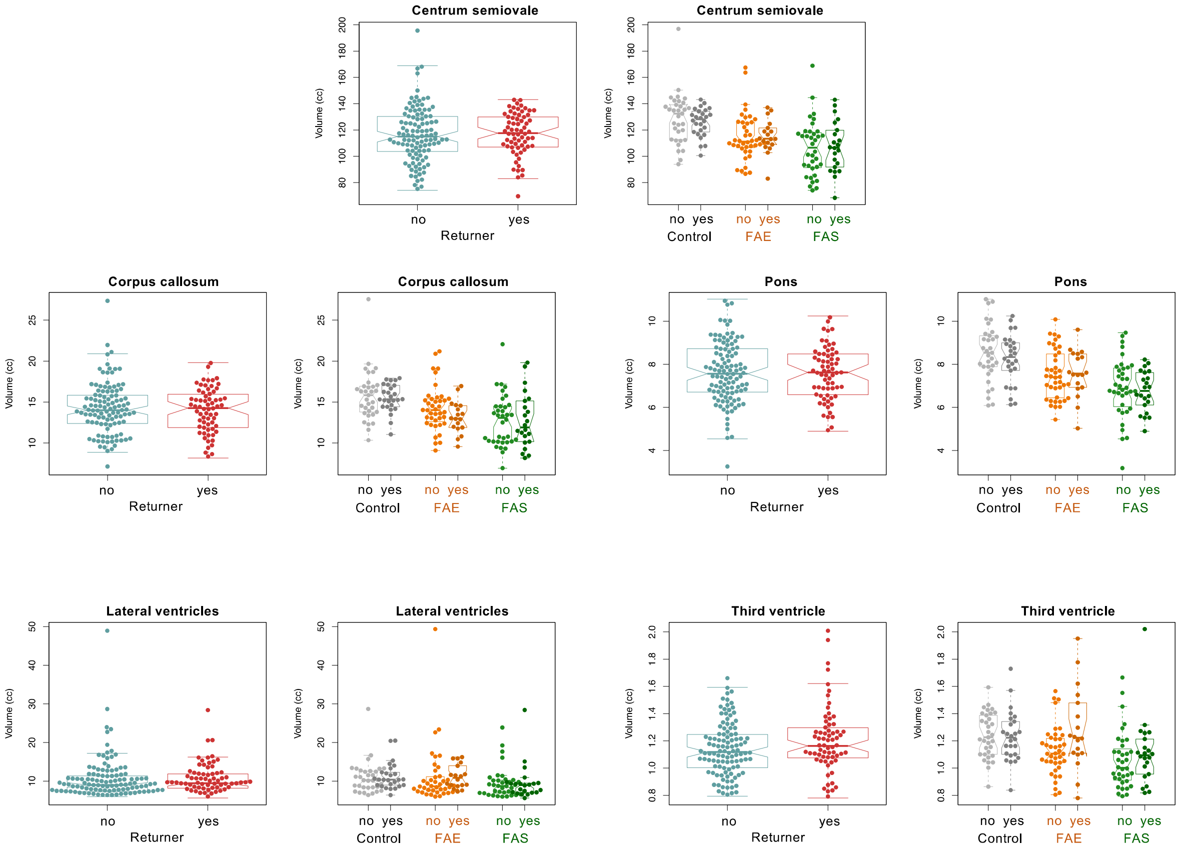

eFigure 3

© 2023 Pfefferbaum A et al. *JAMA Network Open.*

Group Differences between Non-returners and Returners

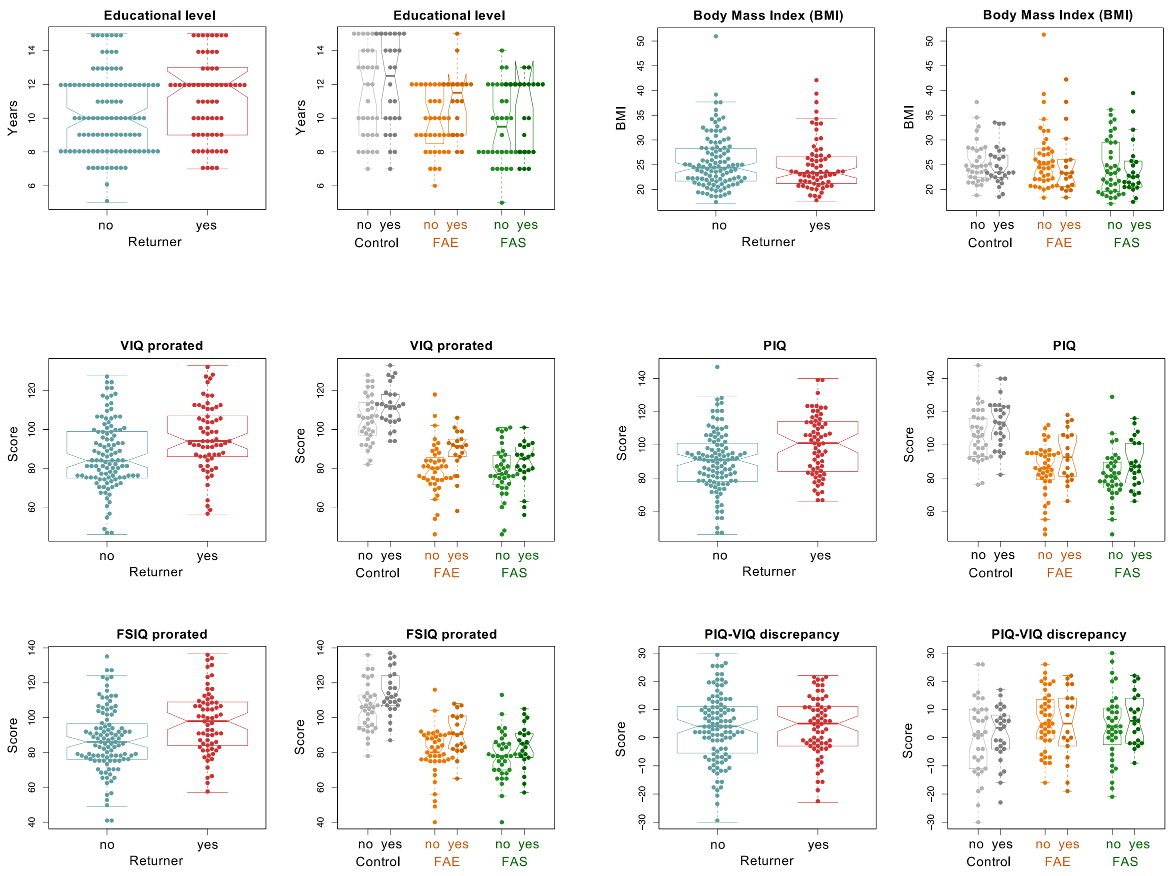

eFigure 4

© 2023 Pfefferbaum A et al. *JAMA Network Open.*

Group Differences in Cortical Gray Matter Volumes

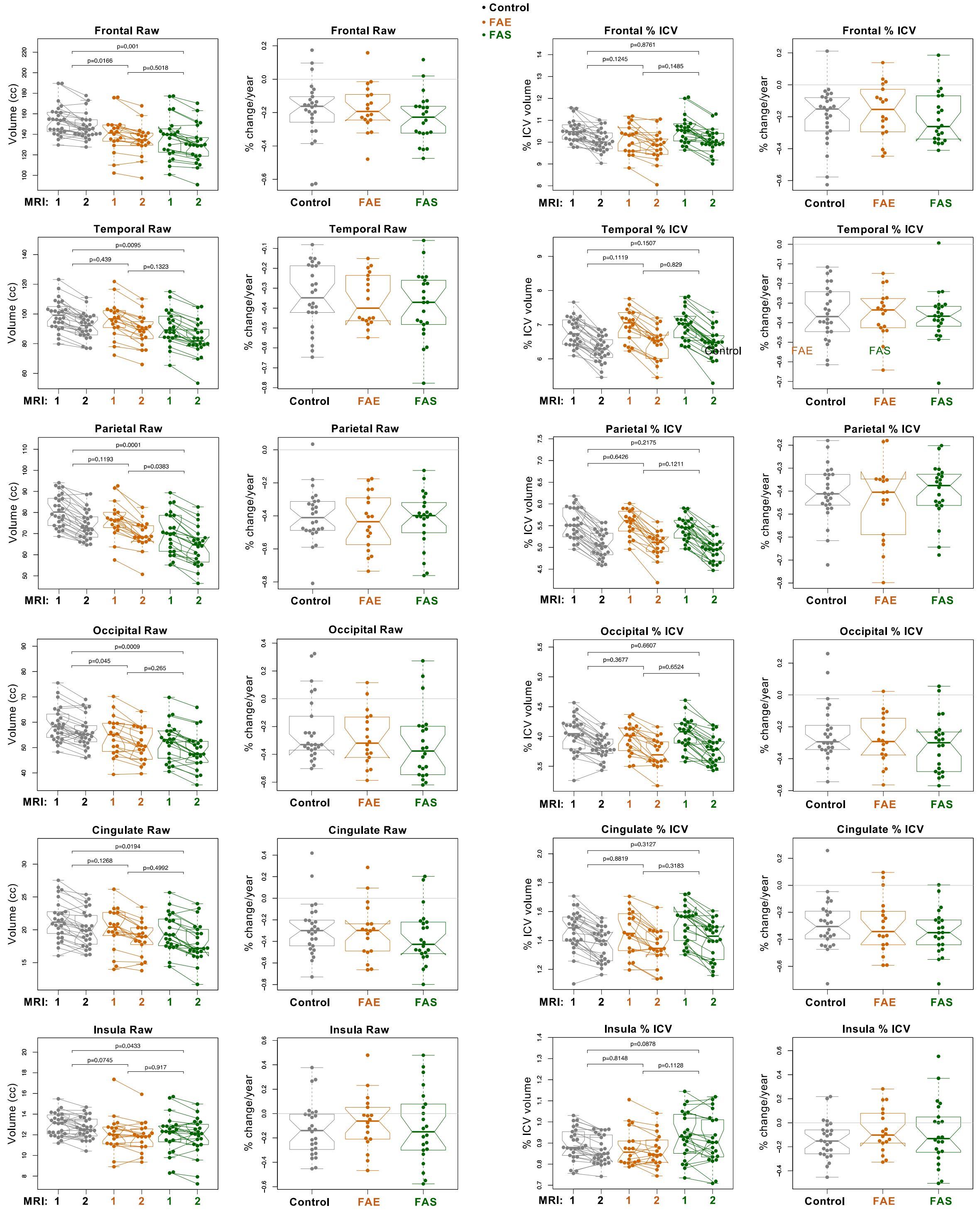

eFigure 5

© 2023 Pfefferbaum A et al. *JAMA Network Open.*

Group Differences in White Matter Volumes

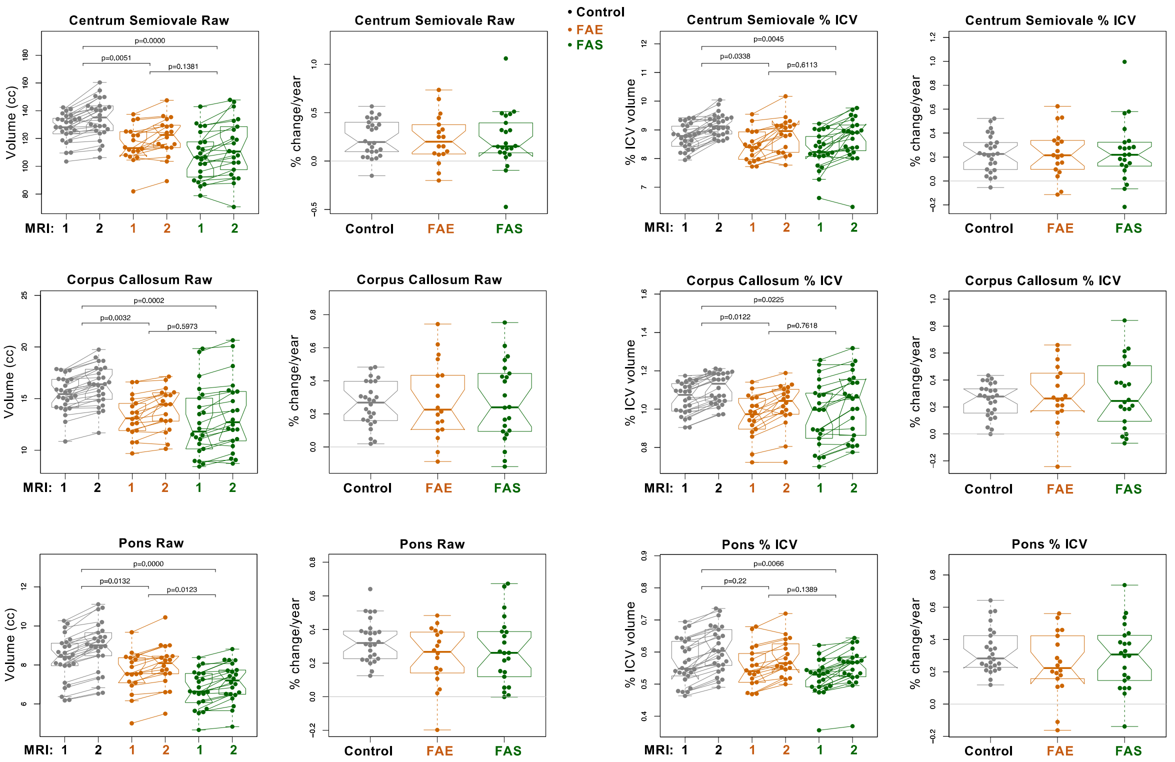

eFigure 6

© 2023 Pfefferbaum A et al. *JAMA Network Open.*

Group Differences in Cerebellar Volumes

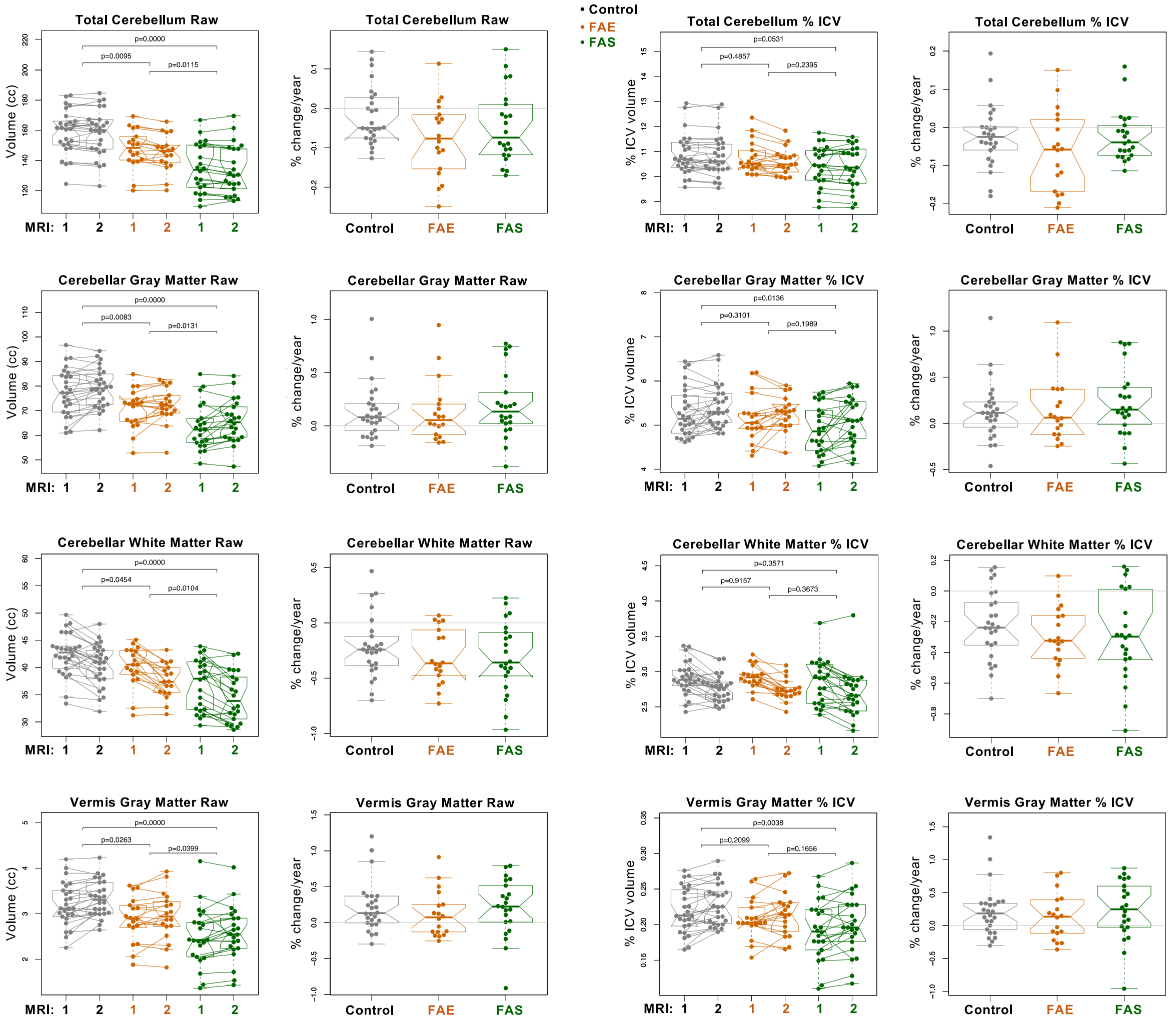

eFigure 7

© 2023 Pfefferbaum A et al. *JAMA Network Open.*

eFigure Captions

eFigure 1. Boxplots and beeswarm plots of intracranial volume (ICV) values for all returning vs. non-returning participants (left) and by group.

eFigure 2. Pairs of boxplots and beeswarm plots of volumes of each cortical region for all returning vs. non-returning participants (left) and by group (right).

eFigure 3. Pairs of boxplots and beeswarm plots of volumes of white matter and ventricular measures for all returning vs. non-returning participants (left) and by group (right).

eFigure 4. Pairs of boxplots and beeswarm plots of demographic data for all returning vs. non-returning participants (left) and by group (right). VIQ=Verbal Intelligence Quotient; PIQ=Performance Intelligence Quotient; FSIQ=Full Scale Intelligence Quotient.

eFigure 5. For each region, the pairs of boxplots on the left are raw (uncorrected) volumes of cortical regions for each MRI session of each participant in the three groups. The pairs of boxplots on the right are the same data expressed as a percent of ICV. The brackets at the top with *P*-values indicate the statistical results of group differences.

eFigure 6. For each region, the pairs of boxplots on the left are raw (uncorrected) volumes of white matter regions for each MRI session of each participant in the three groups. The pairs of boxplots on the right are the same data expressed as a percent of ICV. The brackets at the top with *P*-values indicate the statistical results of group differences.

eFigure 7. For each region, the pairs of boxplots on the left are raw (uncorrected) volumes of cerebellar regions for each MRI session of each participant in the three groups. The pairs of boxplots on the right are the same data expressed as a percent of ICV. The brackets at the top with *P*-values indicate the statistical results of group differences.

© 2023 Pfefferbaum A et al. *JAMA Network Open.*
