# Supplementary material for: Brain Volume in Fetal Alcohol Spectrum Disorders Over a 20-Year Span
Source: JAMA Netw Open. 2023 Nov 17;6(11):e2343618. doi: 10.1001/jamanetworkopen.2023.43618 (PMC10656646; doi:10.1001/jamanetworkopen.2023.43618)
Supplement: Supplement 2. — Data Sharing Statement [file jamanetwopen-e2343618-s002.pdf]

Data Sharing Statement

Pfefferbaum. Brain Volume in Fetal Alcohol Spectrum Disorders Over a 20-Year Span. *JAMA Netw Open*. Published November 17, 2023. doi:10.1001/jamanetworkopen.2023.43618

Data
Data available: No

Additional Information
Explanation for why data not available: Data from this study will not be shared. At the time MRI1 data were acquired, data-sharing agreements were not part of the informed consent process. Furthermore, this work is part of an ongoing longitudinal study on long-term effects of prenatal alcohol and further analyses need to be completed.
